# *tert*-Butyl 2-methyl-2-(4-nitro­benzo­yl)propanoate

**DOI:** 10.1107/S1600536810003119

**Published:** 2010-01-30

**Authors:** Chelsey M. Crosse, Marshall W. Logue, Rudy L. Luck, Louis R. Pignotti, Melissa F. Waineo

**Affiliations:** a Department of Chemistry, 1400 Townsend Drive, Michigan Technological University, Houghton, MI 49931, USA

## Abstract

The title compound, C_15_H_19_NO_5_, is bent with a dihedral angle of 61.8 (2)° between the mean planes of the benzene ring and a group encompassing the ester functionality (O=C—O—C). The dihedral angle of 0.8 (2)° between the mean planes of the nitro group and the benzene ring indicates near coplanarity. In the crystal, each mol­ecule is linked to four adjacent mol­ecules by weak C—H⋯O hydrogen-bonding inter­actions. Both benzene H atoms *ortho* to the ketone O atom form C—H⋯O hydrogen bonds with the keto O atoms of two neighboring mol­ecules (of the keto and ester groups, respectively), and the two other inter­actions involve the H atoms from a methyl group of the dimethyl residue, displaying C—H⋯O inter­actions with the O atoms of the nitro groups. These four inter­actions for each mol­ecule lead to the formation of two-dimensional sheets with a hydro­philic inter­ior, held together by weak hydrogen-bonded inter­actions, and a hydro­phobic exterior composed of protruding methyl groups which interst­ack with the methyl groups in adjacent sheets.

## Related literature

For the synthesis, spectroscopic characterization and reactivity of the title compound, see: Logue (1974 ▶); Logue *et al.* (1975 ▶). For related structures, see: Crosse *et al.* (2010 ▶); Gould *et al.* (2010 ▶); Logue *et al.* (2010 ▶). For the syntheses and characterization of structurally similar indanone-derived β-keto ester derivatives, see: Alemán *et al.* (2007 ▶); Elsner *et al.* (2008 ▶); Mouri *et al.* (2009 ▶); Noritake *et al.* (2008 ▶); Rigby & Dixon (2008 ▶); Wang *et al.* (2006 ▶). For weak hydrogen-bonded inter­actions, see: Karle *et al.* (2009 ▶).
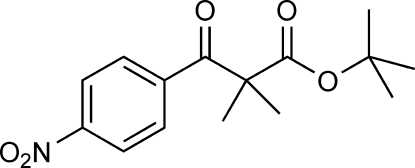


## Experimental

### 

#### Crystal data

C_15_H_19_NO_5_
*M* *_r_* = 293.31
Monoclinic, 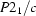

*a* = 11.379 (4) Å
*b* = 11.393 (4) Å
*c* = 12.283 (5) Å
β = 94.88 (3)°
*V* = 1586.6 (10) Å^3^
*Z* = 4
Mo *K*α radiation
μ = 0.09 mm^−1^
*T* = 291 K
0.50 × 0.20 × 0.05 mm

#### Data collection

Enraf–Nonius TurboCAD-4 diffractometer
Absorption correction: ψ scan (North *et al.*, 1968 ▶) *T*_min_ = 0.931, *T*_max_ = 0.993
2933 measured reflections
2785 independent reflections
1196 reflections with *I* > 2σ(*I*)
*R*_int_ = 0.072
3 standard reflections every 166 min  intensity decay: 3%

#### Refinement

*R*[*F* ^2^ > 2σ(*F* ^2^)] = 0.061
*wR*(*F* ^2^) = 0.171
*S* = 0.98
2785 reflections
195 parameters
H-atom parameters constrained
Δρ_max_ = 0.17 e Å^−3^
Δρ_min_ = −0.18 e Å^−3^

### 

Data collection: *CAD-4 EXPRESS* (Enraf–Nonius, 1994 ▶); cell refinement: *CAD-4 EXPRESS*; data reduction: *XCAD4* (Harms & Wocadlo, 1995 ▶); program(s) used to solve structure: *SIR2004* (Burla *et al.*, 2005 ▶); program(s) used to refine structure: *SHELXL97* (Sheldrick, 2008 ▶); molecular graphics: *ORTEP-3 for Windows* (Farrugia, 1997 ▶) and *Mercury* (Macrae *et al.*, 2008 ▶); software used to prepare material for publication: *WinGX* (Farrugia, 1999 ▶) and *publCIF* (Westrip, 2010 ▶).

## Supplementary Material

Crystal structure: contains datablocks global, I. DOI: 10.1107/S1600536810003119/zl2264sup1.cif

Structure factors: contains datablocks I. DOI: 10.1107/S1600536810003119/zl2264Isup2.hkl

Additional supplementary materials:  crystallographic information; 3D view; checkCIF report

## Figures and Tables

**Table 1:** Hydrogen-bond geometry (Å, °)

*D*—H⋯*A*	*D*—H	H⋯*A*	*D*⋯*A*	*D*—H⋯*A*
C3—H3⋯O3^i^	0.93	2.51	3.191 (4)	130
C5—H5⋯O4^ii^	0.93	2.57	3.387 (5)	147
C10—H10*B*⋯O2^iii^	0.96	2.71	3.535 (5)	145

Symmetry codes: (i) 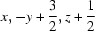; (ii) 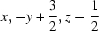; (iii) 
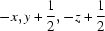
.

**Fig. 1.**
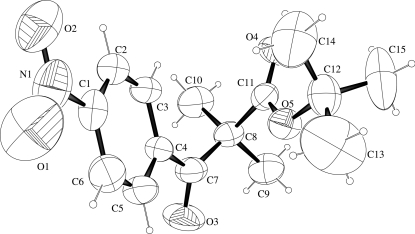
ORTEP-3 (Farrugia, 1997) drawing of the title compound with displacement ellipsoids drawn at the 50% probability level.

**Fig. 2.**
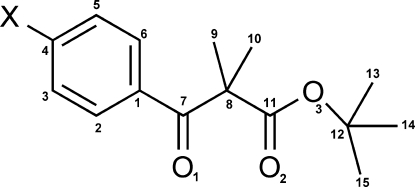
Labelling of the atoms for the purposes of defining the equivalent torsional angles in Table 2.

**Fig. 3.**
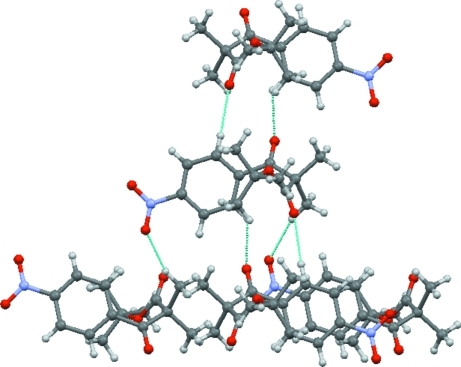
A Mercury (Macrae et al., 2008) illustration of the packing of the title compound depicting the H-bonded linkages to four adjacent molecules using blue dashed lines.

**Fig. 4.**
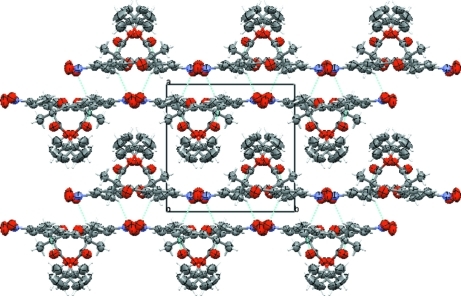
An expansion of the unit cell packing produced with Mercury (Macrae et al., 2008) depicting the nature of the weak H-bonded interactions illustrated with blue dashed lines and the relationships between non-interacting sheets looking down the c-axis.

**Fig. 5.**
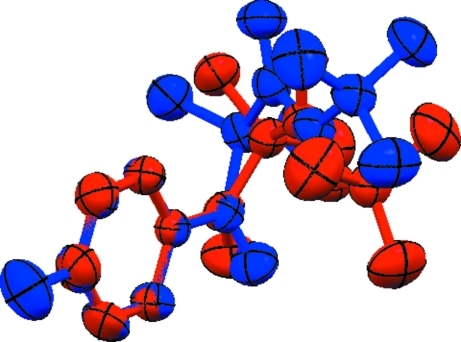
Overlay of molecules produced with Mercury (Macrae et al., 2008) with H– (red), (Logue et al., 2010) and CH3– (blue), (Gould et al., 2010) as substituents on the para position of the phenyl ring.

**Fig. 6.**
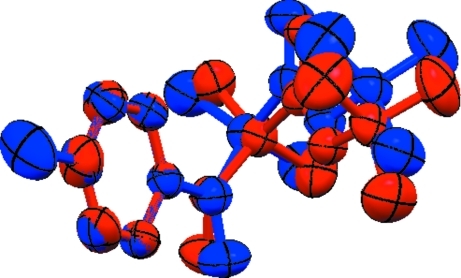
Overlay of molecules produced with Mercury (Macrae et al., 2008) with H–(red), (Logue et al., 2010) and Cl–(blue), (Crosse et al., 2010) as substituents on the para position of the phenyl ring.

**Fig. 7.**
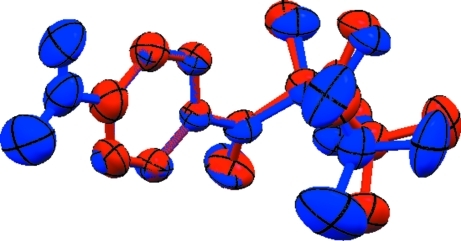
Overlay of molecules produced with Mercury (Macrae et al., 2008) with H–(red), (Logue et al., 2010) as the substituent on the para position of the phenyl ring and the title compound (blue).

**Fig. 8.**
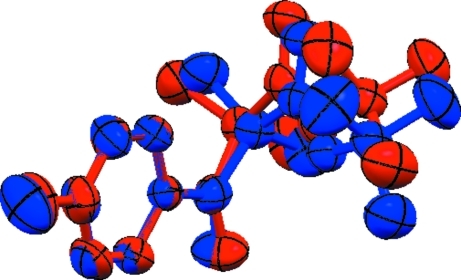
Overlay of almost isosteric molecules produced with Mercury (Macrae et al., 2008) with CH3–(red), (Gould et al., 2010) and Cl–(blue), (Crosse et al., 2010) as substituents on the para position of the phenyl ring.

**Fig. 9.**
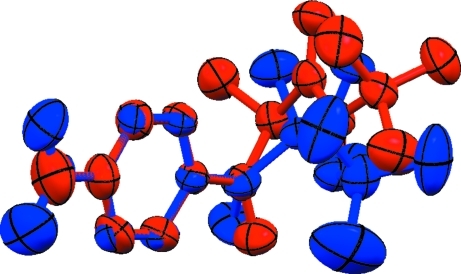
Overlay of almost isosteric molecules produced with Mercury (Macrae et al., 2008) with CH~3~–(red), (Gould et al., 2010) as the substituent on the para position of the phenyl ring and the title compound (blue).

**Fig. 10.**
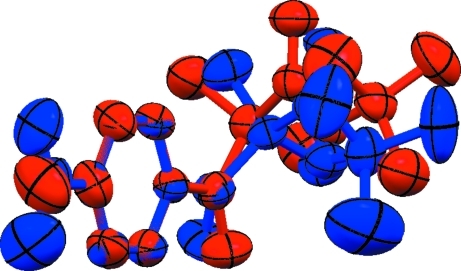
Overlay of almost isosteric molecules produced with Mercury (Macrae et al., 2008) with Cl–(red), (Crosse et al., 2010) as the substituent on the para position of the phenyl ring and the title compound (blue).

## supplementary crystallographic information

### Comment

Treatment of 2,2-disubstituted *t*-butyl β-keto esters with trifluoroacetic acid at room temperature quantitatively generates the corresponding 2,2-disubstituted β-keto acids, which were used to probe the nature of the transition state of their thermal decarboxylation (Logue *et al.*, 1975). Structurally similar indanone-derived β-keto ester derivatives have been prepared recently (Alemán *et al.*, 2007; Elsner *et al.*, 2008; Mouri *et al.*, 2009; Noritake *et al.*, 2008; Rigby & Dixon, 2008; Wang *et al.*, 2006). The directing nature of weak C—H···O H-bonds has been noted to be of importance to afford the three dimensional structure observed in these kinds of molecules (Karle *et al.*, 2009).

In this contribution we present the solid state structure of one such 2,2-disubstituted β-keto acid, i.e. the title compound being the 4-nitrophenyl derivative, and its structure is compared with those of a series of other such compounds with different substitutents in the *para*-position of the phenyl ring.

The title compound, C_15_H_19_N_1_O_5_, which is the final compound in a series of four (Crosse *et al.*, 2010; Gould *et al.*, 2010; Logue *et al.*, 2010), is bent and has a dihedral angle between the mean planes of the phenyl ring and the ester functionality of 61.8 (2)°. The low value of the dihedral angle, 0.8 (2)°, between the mean planes for the nitro group and the phenyl ring suggests coplanarity. Each molecule is bonded to four adjacent molecules by weak C—H···O hydrogen bonding interactions. Both phenyl H-atoms *ortho* to the ketone O-atom form C—H···O hydrogen bonds with keto O atoms of two neighboring molecules (of the keto and ester groups, respectively), and the two other interactions involve H-atoms on the dimethyl group displaying C—H···O interactions with the O-atoms on the nitro groups, Fig. 3. These four interactions for each molecule result in two-dimensional sheets with a hydrophilic interior held together by weak H-bonded interactions and a hydrophobic exterior composed of protruding methyl groups (from the *t*-butyl moiety) which interact with the same on adjacent sheets, Fig. 4. This arrangement seems to result in a more tightly packed arrangement of molecules than in the other three derivatives, which without the nitro group as another acceptor for C—H···O interactions form only C—H···O bonded chains rather than layers and this might also be the reason why the density at 1.228 g/cm^-3^ is greatest for the title compound compared to the other three molecules which range from 1.108—1.194 g/cm^-3^.

The four structures studied which differ only in the substituent on the *para*-position of the phenyl ring of the title compound, H–, (Logue *et al.*, 2010), CH_3_–, (Gould *et al.*, 2010), Cl-, (Crosse *et al.*, 2010), and NO_2_–, (this paper) are structurally very similar and there are no significant differences in the bonding distances or bond angle measurements in these four complexes. They do however display some conformational flexibility which are expressed by the slight variations of the torsion angles of the *t*-butyl oxopropanoate unit, listed in Table 2 (the common atom numbering used for all four atoms is given in Figure 2). Individually these angles vary by only small amounts, the largest variation is observed for 2—1—7—8 (the torsion angle between the phenyl ring and the plane of the keto functionality) wihich differs by 35° between the NO_2_- and the CH_3_- compound with -154.6 (3) and +170.4 (2) °, respectively. The maximum deviation for the next two torsion angles along the C(=O)—C—C(=O)—O—C backbone are with 17.22 (for 1—7—8—11) and 11.42 (for 7—8—11—O3) still significant. The last of these four torsion angles (8—11—O3—12), shows no significant variation between the four compounds.

The similarity of the torsion angles leads for all four compounds to a similar molecular geometry. They are all bent and the *t*-butyl 2,2-dimethyl units are folding back on the phenyl moiety with angles between the two units between 61.8 (2) and 75.3 (1)° at the extreme for the NO_2_– and CH_3_– substituted derivatives. The variations of the torsional angles result however in slightly different conformations for the four molecules, as can be seen in overlays between the various molecules as shown in Figs. 5 to 10. In all figures the six phenyl C-atoms in the two molecules depicted were positioned on top of one another in the overlay using the program Mercury (Macrae *et al.*,  2008). The figures depict overlays of the H–derivative (in red) with one of the molecules containing a CH_3_–substituent Fig. 5, Cl- Fig. 6 and NO_2_– group Fig. 7 (each in blue); the CH_3_–substituent (red) overlayed with Cl- (blue), Fig. 8 and the NO_2_–group (blue), Fig. 9 and the Cl– (red) and NO_2_–derivative (blue) overlay, Fig 10.

The effect of the torsional angle defined as 2—1—7—8 is then apparent as this displays the greatest variation among the other selected torsional angles listed in Table 2. As discussed above, the greatest difference in this angle occurs between the CH_3_– and the NO_2_–derivative, Fig. 9, and in this drawing the *t*-butyl groups display the greatest separation compared to the others. On the other hand the overlay displayed in Fig. 7 featuring the H– and NO_2_– derivatives show the largest overlap between the two molecules with equivalent 2—1—7—8 torsional angles of -160.6 (2) and -154.6 (3)°.

These different conformations and the resultant shapes necessarily lead to different packing of the molecules, caused by different combinations of weak C-H···O interactions. These result in infinite chains of vastly different linkage arrangements in the case of the H–, (Logue *et al.*, 2010), CH_3_–, (Gould *et al.*, 2010), and Cl-, (Crosse *et al.*, 2010), derivatives and a 2-dimensional sheet for the NO_2_– derivative (this paper). One constant theme in all of these structures is the acidity of one of the hydrogen atoms in the dimethyl moiety as one of the H atoms is involved in hydrogen bonding in all of them as is the case for other β-keto esters (Alemán *et al.*, 2007; Elsner *et al.*, 2008; Mouri *et al.*, 2009; Noritake *et al.*, 2008; Rigby & Dixon, 2008; Wang *et al.*, 2006).

### Experimental

Crystals of the material were synthesized as reported earlier and were grown by evaporation of a solution in hexane (Logue, 1974). M.p. 605-610 K. IR (KBr pellet, cm^-1^): 3113, 2986, 2937 (m, C—H), 1734 (v.s., ester C=O), 1687 (v.s., ketone C=O) 1601 (m, C—C), 1524 (v.s., NO_2_), 1458 (*m*), 1389 (*m*), 1369 (*s*), 1345 (v.s., NO_2_), 1282 (s, alkyl methyl C—H), 1254 (*s*), 1152 (v.s., ester C—O), 989 (*s*), 932 (*m*), 847 (s, C—H bend), 721 (*s*). ^1^H NMR (CDCl_3_) δ: 1.28 (s, 9*H*), 1.50 (s, 6*H*), 7.99 (d, 2*H*, J=9.2 Hz), 8.24 (d, 2*H*, J=9.2 Hz). ^13^C NMR (CDCl_3_) δ: 23.8, 27.8, 54.6, 82.6, 129.9, 130.9, 140.5, 150.1, 173.4, 196.9.

### Refinement

All H atoms were placed at calculated positions, with C—H = 0.93 Å (aromatic) or 0.96 Å (methyl) and refined using a riding model with *U*_iso_(H) constrained to be 1.5 *U*_eq_(C) for methyl groups and 1.2 *U*_eq_(C) for all other C atoms. The quality of the data as reflected by only 66% of the reflections observed, large ADP's and inaccurate C—C bond lengths is low. The data had been collected on a 30 year old single point detector instrument not equipped with a low temperature device as part of a class project with undergraduate students. Due to the time constraints imposed by the class schedule a maximum exposure time of 60 s had to be alloted for measuring each reflection.

### Crystal data

**Table:**

C_15_H_19_NO_5_	*F*(000) = 624
*M**_r_* = 293.31	*D*_x_ = 1.228 Mg m^−^^3^
Monoclinic, *P*2_1_/*c*	Mo *K*α radiation, λ = 0.71073 Å
Hall symbol: -P 2ybc	Cell parameters from 25 reflections
*a* = 11.379 (4) Å	θ = 10–15°
*b* = 11.393 (4) Å	µ = 0.09 mm^−^^1^
*c* = 12.283 (5) Å	*T* = 291 K
β = 94.88 (3)°	Prism, colourless
*V* = 1586.6 (10) Å^3^	0.50 × 0.20 × 0.05 mm
*Z* = 4	

### Data collection

**Table:**

Enraf–Nonius TurboCAD-4 diffractometer	1196 reflections with *I* > 2σ(*I*)
Radiation source: Enraf Nonius FR590	*R*_int_ = 0.072
graphite	θ_max_ = 25.0°, θ_min_ = 1.8°
non–profiled ω/2τ scans	*h* = 0→13
Absorption correction: ψ scan (North *et al.*, 1968)	*k* = 0→13
*T*_min_ = 0.931, *T*_max_ = 0.993	*l* = −14→14
2933 measured reflections	3 standard reflections every 166 min
2785 independent reflections	intensity decay: 3%

### Refinement

**Table:**

Refinement on *F*^2^	Primary atom site location: structure-invariant direct methods
Least-squares matrix: full	Secondary atom site location: difference Fourier map
*R*[*F*^2^ > 2σ(*F*^2^)] = 0.061	Hydrogen site location: inferred from neighbouring sites
*wR*(*F*^2^) = 0.171	H-atom parameters constrained
*S* = 0.98	*w* = 1/[σ^2^(*F*_o_^2^) + (0.0756*P*)^2^] where *P* = (*F*_o_^2^ + 2*F*_c_^2^)/3
2785 reflections	(Δ/σ)_max_ < 0.001
195 parameters	Δρ_max_ = 0.17 e Å^−^^3^
0 restraints	Δρ_min_ = −0.18 e Å^−^^3^

### Special details

**Table:**

Experimental. (North *et al.*, 1968) Number of psi-scan sets used was 2. Theta correction was applied. Averaged transmission function was used. No Fourier smoothing was applied.
Geometry. All s.u.'s (except the s.u. in the dihedral angle between two l.s. planes) are estimated using the full covariance matrix. The cell s.u.'s are taken into account individually in the estimation of s.u.'s in distances, angles and torsion angles; correlations between s.u.'s in cell parameters are only used when they are defined by crystal symmetry. An approximate (isotropic) treatment of cell s.u.'s is used for estimating s.u.'s involving l.s. planes.
Refinement. Refinement of *F*^2^ against ALL reflections. The weighted *R*-factor *wR* and goodness of fit *S* are based on *F*^2^, conventional *R*-factors *R* are based on *F*, with *F* set to zero for negative *F*^2^. The threshold expression of *F*^2^ > 2σ(*F*^2^) is used only for calculating *R*-factors(gt) *etc*. and is not relevant to the choice of reflections for refinement. *R*-factors based on *F*^2^ are statistically about twice as large as those based on *F*, and *R*- factors based on ALL data will be even larger.

### Fractional atomic coordinates and isotropic or equivalent isotropic displacement parameters (Å^2^)

**Table:**

	*x*	*y*	*z*	*U*_iso_*/*U*_eq_	
N1	0.1227 (3)	0.3124 (4)	0.1368 (4)	0.0837 (12)	
O1	0.1312 (4)	0.2370 (3)	0.0679 (4)	0.1350 (15)	
O2	0.1050 (3)	0.2912 (3)	0.2318 (3)	0.1021 (11)	
C1	0.1353 (3)	0.4356 (3)	0.1031 (3)	0.0577 (10)	
C2	0.1269 (3)	0.5237 (4)	0.1783 (3)	0.0604 (11)	
H2	0.1122	0.5059	0.2498	0.073*	
C3	0.1404 (3)	0.6375 (4)	0.1472 (3)	0.0564 (10)	
H3	0.1357	0.6976	0.198	0.068*	
C4	0.1613 (3)	0.6645 (3)	0.0391 (3)	0.0502 (9)	
C5	0.1682 (3)	0.5738 (4)	−0.0348 (3)	0.0624 (11)	
H5	0.181	0.5911	−0.1068	0.075*	
C6	0.1564 (3)	0.4586 (4)	−0.0042 (3)	0.0679 (12)	
H6	0.1625	0.3979	−0.054	0.082*	
C7	0.1776 (3)	0.7879 (4)	−0.0005 (3)	0.0570 (10)	
O3	0.1533 (3)	0.8083 (3)	−0.0971 (2)	0.0886 (10)	
C8	0.2210 (3)	0.8869 (3)	0.0759 (3)	0.0506 (9)	
C9	0.2737 (4)	0.9859 (3)	0.0097 (3)	0.0721 (12)	
H9A	0.3002	1.0485	0.058	0.108*	
H9B	0.2145	1.015	−0.044	0.108*	
H9C	0.3391	0.9559	−0.0262	0.108*	
C10	0.1165 (3)	0.9360 (4)	0.1339 (3)	0.0759 (13)	
H10A	0.142	1.003	0.1769	0.114*	
H10B	0.088	0.8766	0.1806	0.114*	
H10C	0.0543	0.9587	0.0803	0.114*	
C11	0.3195 (3)	0.8458 (3)	0.1591 (3)	0.0480 (9)	
O4	0.3323 (2)	0.8769 (2)	0.2523 (2)	0.0727 (8)	
O5	0.3918 (2)	0.7753 (2)	0.11076 (18)	0.0583 (7)	
C12	0.4942 (3)	0.7164 (4)	0.1705 (4)	0.0714 (12)	
C13	0.5390 (6)	0.6385 (6)	0.0848 (5)	0.165 (3)	
H13A	0.4792	0.5827	0.0607	0.248*	
H13B	0.6081	0.5976	0.1148	0.248*	
H13C	0.5586	0.6854	0.0239	0.248*	
C14	0.4540 (5)	0.6455 (4)	0.2639 (5)	0.123 (2)	
H14A	0.3995	0.5862	0.2359	0.184*	
H14B	0.4158	0.6961	0.3124	0.184*	
H14C	0.5209	0.6088	0.3028	0.184*	
C15	0.5827 (4)	0.8075 (5)	0.2131 (5)	0.134 (2)	
H15A	0.6016	0.8576	0.1542	0.2*	
H15B	0.6531	0.7692	0.2438	0.2*	
H15C	0.5498	0.8536	0.2684	0.2*	

### Atomic displacement parameters (Å^2^)

**Table:**

	*U*^11^	*U*^22^	*U*^33^	*U*^12^	*U*^13^	*U*^23^
N1	0.061 (2)	0.069 (3)	0.124 (4)	0.001 (2)	0.023 (2)	−0.001 (3)
O1	0.176 (4)	0.063 (2)	0.174 (4)	0.011 (2)	0.062 (3)	−0.010 (2)
O2	0.102 (3)	0.086 (2)	0.121 (3)	−0.008 (2)	0.025 (2)	0.028 (2)
C1	0.043 (2)	0.050 (3)	0.080 (3)	−0.005 (2)	0.005 (2)	0.003 (2)
C2	0.064 (3)	0.069 (3)	0.049 (2)	−0.013 (2)	0.0057 (19)	−0.002 (2)
C3	0.066 (3)	0.065 (3)	0.039 (2)	−0.011 (2)	0.0061 (18)	−0.0035 (19)
C4	0.046 (2)	0.069 (3)	0.0356 (19)	−0.0047 (19)	0.0026 (16)	−0.003 (2)
C5	0.063 (3)	0.081 (3)	0.044 (2)	0.000 (2)	0.0116 (19)	−0.006 (2)
C6	0.058 (3)	0.076 (3)	0.071 (3)	0.004 (2)	0.015 (2)	−0.018 (2)
C7	0.061 (2)	0.070 (3)	0.041 (2)	0.003 (2)	0.0055 (18)	0.007 (2)
O3	0.127 (3)	0.096 (2)	0.0395 (16)	−0.0103 (19)	−0.0110 (16)	0.0177 (15)
C8	0.057 (2)	0.052 (2)	0.0442 (19)	0.0064 (19)	0.0082 (18)	0.0069 (18)
C9	0.091 (3)	0.058 (3)	0.068 (3)	0.003 (2)	0.009 (2)	0.017 (2)
C10	0.068 (3)	0.089 (3)	0.071 (3)	0.029 (3)	0.012 (2)	0.007 (2)
C11	0.050 (2)	0.047 (2)	0.047 (2)	−0.0044 (19)	0.0103 (19)	−0.0005 (19)
O4	0.0762 (19)	0.093 (2)	0.0472 (16)	0.0111 (16)	−0.0022 (13)	−0.0140 (15)
O5	0.0554 (16)	0.0646 (16)	0.0550 (15)	0.0147 (14)	0.0055 (13)	−0.0037 (13)
C12	0.050 (2)	0.069 (3)	0.094 (3)	0.012 (2)	−0.002 (2)	0.004 (3)
C13	0.148 (6)	0.181 (6)	0.164 (6)	0.102 (5)	−0.002 (5)	−0.049 (5)
C14	0.096 (4)	0.098 (4)	0.172 (5)	0.012 (3)	0.000 (4)	0.071 (4)
C15	0.059 (3)	0.127 (5)	0.210 (6)	−0.021 (3)	−0.021 (4)	0.015 (5)

### Geometric parameters (Å, °)

**Table:**

N1—O1	1.216 (5)	C9—H9B	0.96
N1—O2	1.225 (4)	C9—H9C	0.96
N1—C1	1.474 (5)	C10—H10A	0.96
C1—C2	1.373 (5)	C10—H10B	0.96
C1—C6	1.384 (5)	C10—H10C	0.96
C2—C3	1.365 (5)	C11—O4	1.195 (4)
C2—H2	0.93	C11—O5	1.326 (4)
C3—C4	1.403 (4)	O5—C12	1.483 (4)
C3—H3	0.93	C12—C13	1.499 (6)
C4—C5	1.381 (5)	C12—C14	1.506 (6)
C4—C7	1.505 (5)	C12—C15	1.509 (6)
C5—C6	1.375 (5)	C13—H13A	0.96
C5—H5	0.93	C13—H13B	0.96
C6—H6	0.93	C13—H13C	0.96
C7—O3	1.218 (4)	C14—H14A	0.96
C7—C8	1.523 (5)	C14—H14B	0.96
C8—C11	1.525 (5)	C14—H14C	0.96
C8—C9	1.541 (5)	C15—H15A	0.96
C8—C10	1.543 (5)	C15—H15B	0.96
C9—H9A	0.96	C15—H15C	0.96
			
O1—N1—O2	123.6 (4)	H9B—C9—H9C	109.5
O1—N1—C1	117.5 (4)	C8—C10—H10A	109.5
O2—N1—C1	118.9 (4)	C8—C10—H10B	109.5
C2—C1—C6	122.0 (4)	H10A—C10—H10B	109.5
C2—C1—N1	119.5 (4)	C8—C10—H10C	109.5
C6—C1—N1	118.5 (4)	H10A—C10—H10C	109.5
C3—C2—C1	119.4 (4)	H10B—C10—H10C	109.5
C3—C2—H2	120.3	O4—C11—O5	125.4 (3)
C1—C2—H2	120.3	O4—C11—C8	125.0 (3)
C2—C3—C4	120.3 (4)	O5—C11—C8	109.5 (3)
C2—C3—H3	119.8	C11—O5—C12	122.9 (3)
C4—C3—H3	119.8	O5—C12—C13	102.7 (3)
C5—C4—C3	118.8 (4)	O5—C12—C14	110.0 (3)
C5—C4—C7	118.1 (3)	C13—C12—C14	111.2 (4)
C3—C4—C7	123.1 (3)	O5—C12—C15	109.5 (3)
C6—C5—C4	121.5 (4)	C13—C12—C15	113.2 (5)
C6—C5—H5	119.3	C14—C12—C15	110.0 (4)
C4—C5—H5	119.3	C12—C13—H13A	109.5
C5—C6—C1	118.0 (4)	C12—C13—H13B	109.5
C5—C6—H6	121	H13A—C13—H13B	109.5
C1—C6—H6	121	C12—C13—H13C	109.5
O3—C7—C4	118.0 (3)	H13A—C13—H13C	109.5
O3—C7—C8	119.6 (3)	H13B—C13—H13C	109.5
C4—C7—C8	122.4 (3)	C12—C14—H14A	109.5
C7—C8—C11	111.5 (3)	C12—C14—H14B	109.5
C7—C8—C9	109.7 (3)	H14A—C14—H14B	109.5
C11—C8—C9	106.4 (3)	C12—C14—H14C	109.5
C7—C8—C10	109.3 (3)	H14A—C14—H14C	109.5
C11—C8—C10	110.7 (3)	H14B—C14—H14C	109.5
C9—C8—C10	109.2 (3)	C12—C15—H15A	109.5
C8—C9—H9A	109.5	C12—C15—H15B	109.5
C8—C9—H9B	109.5	H15A—C15—H15B	109.5
H9A—C9—H9B	109.5	C12—C15—H15C	109.5
C8—C9—H9C	109.5	H15A—C15—H15C	109.5
H9A—C9—H9C	109.5	H15B—C15—H15C	109.5

### Hydrogen-bond geometry (Å, °)

**Table:**

*D*—H···*A*	*D*—H	H···*A*	*D*···*A*	*D*—H···*A*
C3—H3···O3^i^	0.93	2.51	3.191 (4)	130
C5—H5···O4^ii^	0.93	2.57	3.387 (5)	147
C10—H10B···O2^iii^	0.96	2.71	3.535 (5)	145

Symmetry codes: (i) *x*, −*y*+3/2, *z*+1/2; (ii) *x*, −*y*+3/2, *z*−1/2; (iii) −*x*, *y*+1/2, −*z*+1/2.

### Table 2 Comparative torsion angle parameters (°) for related complexes, see Fig. 2 for the definition of atomic labels on the molecules^a^

**Table:**

Torsion	X=H^b^	X=CH_3_^c^	X=Cl^d^	X=NO_2_^e^
2—1—7—8	-160.6 (2)	170.4 (2)	-175.6 (3)	-154.6 (3)
1—7—8—11	43.8 (3)	57.8 (3)	52.8 (3)	40.6 (4)
7—8—11—O3	45.9 (2)	34.1 (3)	41.4 (3)	40.6 (4)
8—11—O3—12	179.50 (17)	176.60 (19)	179.5 (2)	177.4 (3)

Notes: (a) Torsion angles calculated using PARST (Nardelli, 1995). (b) Logue *et al.* (2010); (c) Gould *et al.* (2010); (d) Crosse *et al.* (2010); (e) this work;
